# An Unexpected Limp: A Pediatric Case of Pott’s Disease and a Literature Review

**DOI:** 10.3390/pathogens15090919

**Published:** 2026-08-31

**Authors:** Giulia Truglio, Giulia Linares, Gianluca Coscia, Valeria Garbo, Giovanni Boncori, Chiara Albano, Sara Ashtari, Alessandra Cuccia, Valentina Frasca Polara, Claudia Colomba

**Affiliations:** 1 Division of Pediatric Infectious Diseases, “G. Di Cristina” Hospital, Azienda di Rilievo Nazionale ad Alta Specializzazione (ARNAS) Civico, 90134 Palermo, Italy; linares.giulia11@gmail.com (G.L.); vali.garbo@gmail.com (V.G.); boncori.giovanni@yahoo.it (G.B.); ch.albano27@gmail.com (C.A.); frascapolara.valentina@gmail.com (V.F.P.); claudia.colomba@libero.it (C.C.); 2 Department of Health Promotion, Maternal and Infant Care, Internal Medicine and Medical Specialties, University of Palermo, 90127 Palermo, Italy; gianluco98@gmail.com (G.C.); saraashtari95@gmail.com (S.A.); alessandra.cuccia92@gmail.com (A.C.)

**Keywords:** Pott’s disease, pediatric, tuberculous spondylodiscitis, paradoxical reaction, IRIS, tuberculosis, pediatric tuberculosis, antitubercular treatment, MSMD, *Mycobacterium tuberculosis*

## Abstract

Pott’s disease, also known as tuberculous spondylodiscitis, accounts for 50% of all forms of skeletal tuberculosis. It is rare in children, especially in low-burden TB countries. We present a case of Pott’s disease with miliary tuberculosis in a 22-month-old child. Furthermore, we provide an exploratory literature review of pediatric cases published in PubMed and Scopus between January 2000 and August 2025, including patients aged 0 to 17 years with confirmed or clinically diagnosed tuberculous spondylodiscitis. Sixty-four cases were included. Most cases (81.3%) involved children from highly endemic countries, particularly India. Median age was 10 years, with a median diagnostic delay of 141.4 days. The most common signs and symptoms were back pain (79.6%), followed by difficulty walking (68.4%). The thoracic spine was the most affected segment (60.3%). Sixty-three received anti-TB therapy, 58% required surgical intervention, and treatment led to overall clinical recovery in 96.9% of cases. Neurological deficits were reported in 46% (paresthesia) and 68.4% (motor weakness), with permanent motor sequelae in 8.1% and persistent spinal deformity (kyphosis) in 14.5%. Our case highlights the importance of considering the suspicion of tuberculous spondylodiscitis, even in low-endemic countries, to achieve early diagnosis and appropriate therapeutic management. Early MRI combined with microbiological confirmation is essential to reduce the risk of severe complications, including neurological sequelae, progressive spinal deformities, and potentially life-threatening outcomes in growing patients.

## 1. Introduction

Tuberculosis (TB) is a contagious infectious disease caused by *Mycobacterium tuberculosis* (MTB), whose clinical manifestations vary widely according to the site of infection and the immune status of the host [1]. Although TB has been known since ancient times, it remains a major global health burden [2]. According to the latest WHO Global Tuberculosis Report, an estimated 10.7 million people developed TB worldwide in 2024, including approximately 1.2 million children and young adolescents [2]. In the same year, an estimated 1.23 million people died from TB, making it the world’s leading cause of death from a single infectious agent and one of the top 10 causes of death worldwide [2]. Pulmonary TB is the most common form of the disease. Among the extrapulmonary forms, osteoarticular TB constitutes approximately 35% of cases, the most common manifestation of which (50%) is TB spondylodiscitis [3], also known as Pott’s disease. TB spondylodiscitis is rare in low-burden countries, while it represents one of the main infectious causes of spondylodiscitis among immigrants from TB endemic areas [2,4]. In the pediatric population, osteoarticular TB originates from lymphohematogenous dissemination with early onset due to inadequate control of the infection, whereas in adults it more commonly results from late post-primary reactivation [3]. In children, diagnosis is often delayed due to its insidious course, its poor recognition in low-endemic countries, and limited access to medical care in endemic areas. For these reasons, most patients present with signs and symptoms of spinal cord compression at the time of diagnosis [5]. In the absence of timely and appropriate therapy, Pott’s disease can lead to serious complications, such as spinal deformity, paraplegia, and even death [6]. We describe a rare case of Pott’s disease in a 22-month-old child and provide a review of the literature to report a comprehensive overview of the epidemiological and clinical features of TB spondylodiscitis in children. Due to its rarity in low-burden settings, pediatric Pott’s disease poses a major diagnostic challenge, often leading to delayed recognition and avoidable neurological morbidity. While single case reports illustrate this complexity, aggregate literature syntheses evaluating pediatric cases over the past two decades remain limited. The clinical severity of our patient, involving miliary dissemination, central nervous system involvement, and a paradoxical reaction, perfectly exemplifies how insidious this diagnosis can be. Consequently, we conducted an updated literature review to synthesize available data, offering clinicians a practical resource to navigate such complex scenarios in both endemic and non-endemic settings.

## 2. Case Report

**Figure 1:**
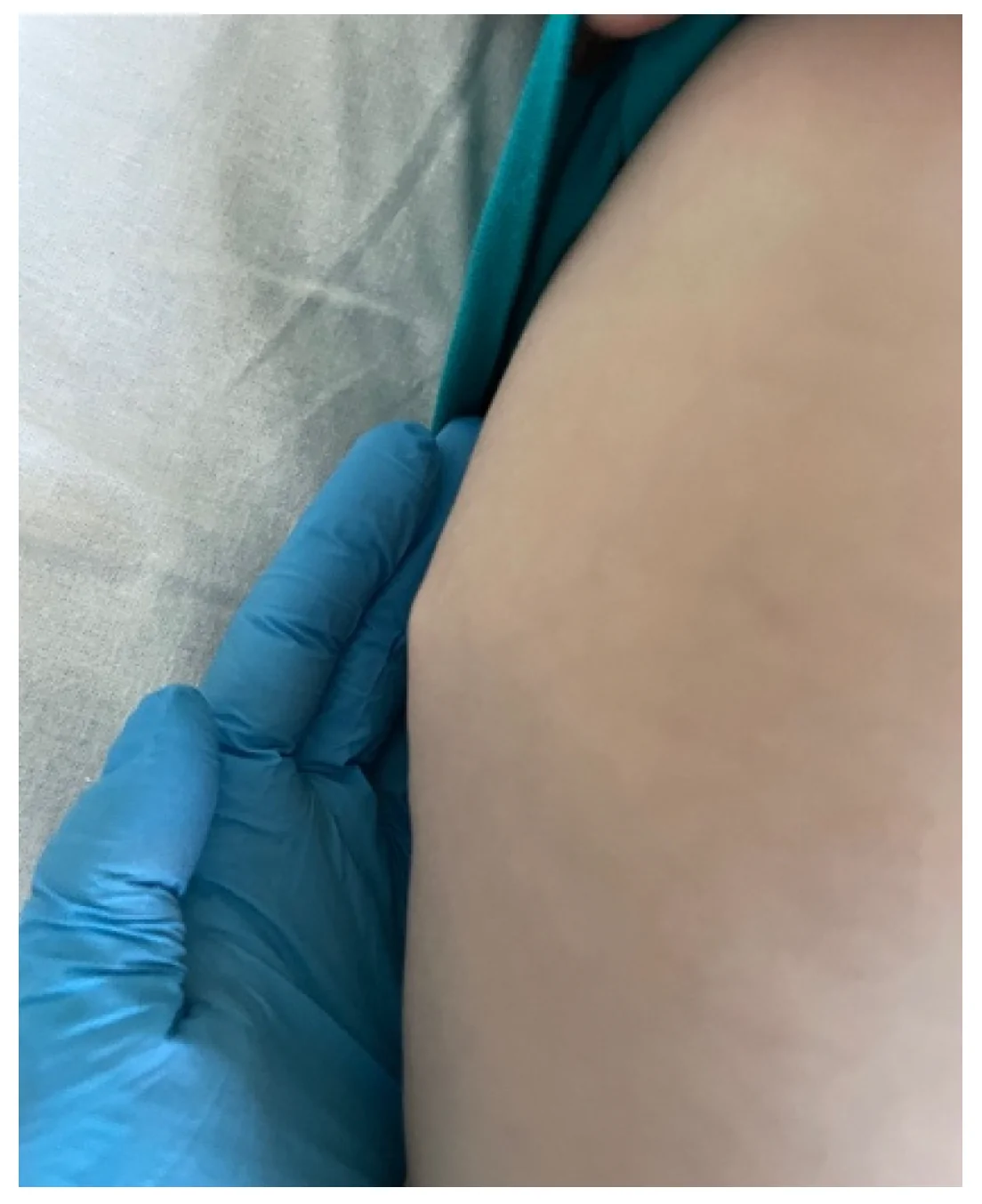
Gibbus deformity of the D10 spinous process.

A 22-month-old boy, born in Italy to Romanian parents, was referred to our centre from another hospital for further diagnostic evaluation. He presented with fever, difficulty walking, and a dorsal swelling, along with a three-month history of weight loss. On admission, the patient was febrile (body temperature, 38.1 °C) and eupneic while breathing room air. Chest auscultation revealed normal breath sounds. Examination of the back showed a deformity over the D10 spinous process. The lesion was fixed to the underlying tissues, firm centrally, and elastic and tender at the periphery, with pink overlying skin (Figure 1).

**Figure 2:**
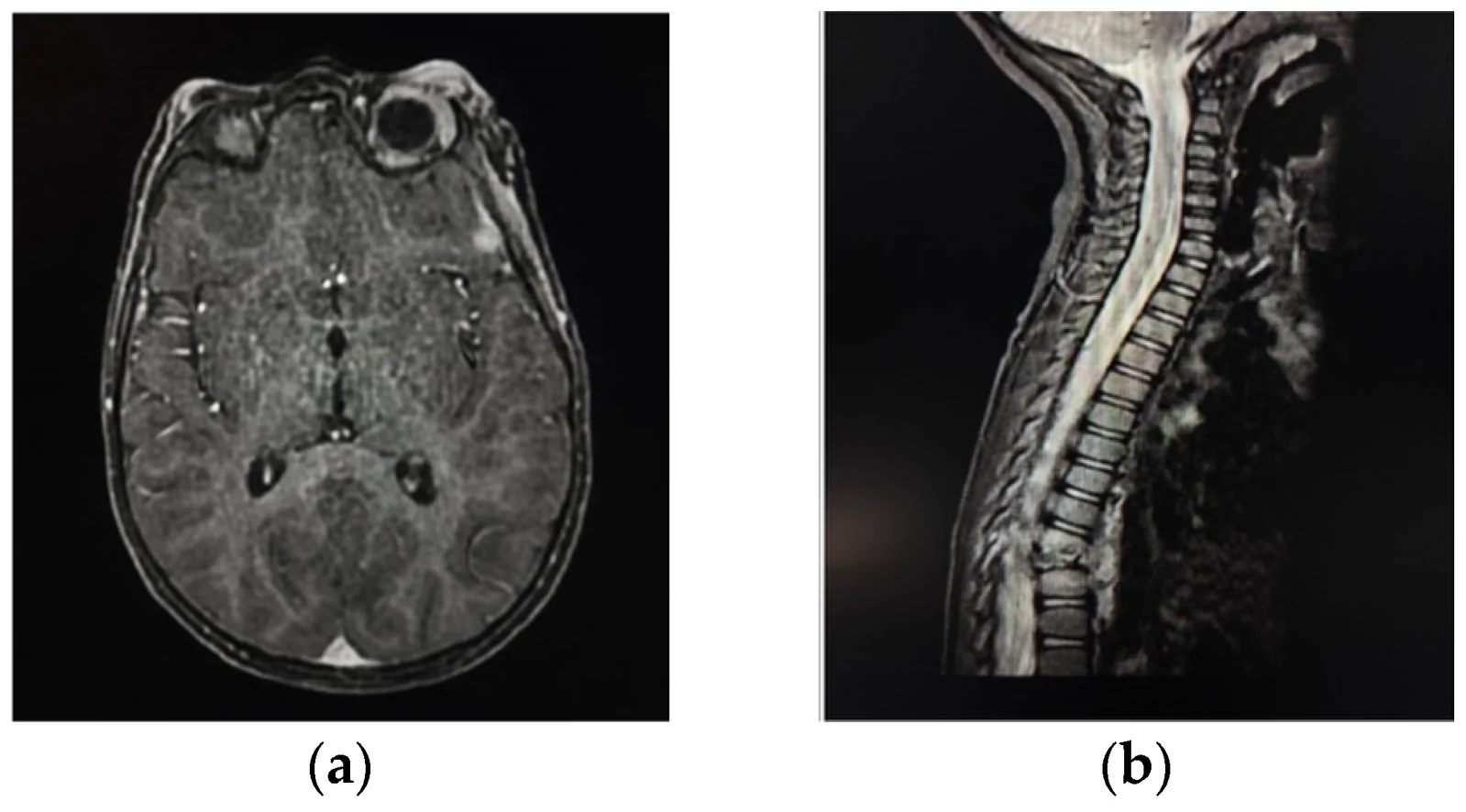
MRI of the brain and spine: (**a**) a hyperintense lesion in the left frontal region; (**b**) erosion and destruction of the D10 vertebral body with involvement of the adjacent intervertebral disc and an associated paravertebral abscess.

Neurological examination was unremarkable, with preserved sensation and full strength and mobility in all four limbs. At the referring hospital, brain and spine magnetic resonance imaging (MRI) revealed a cerebral hyperintense lesion in the left frontal region, erosion of the tenth thoracic vertebral body with destruction of the adjacent intervertebral disc, a paravertebral abscess, and a subpleural collection (Figure 2).

**Figure 3:**
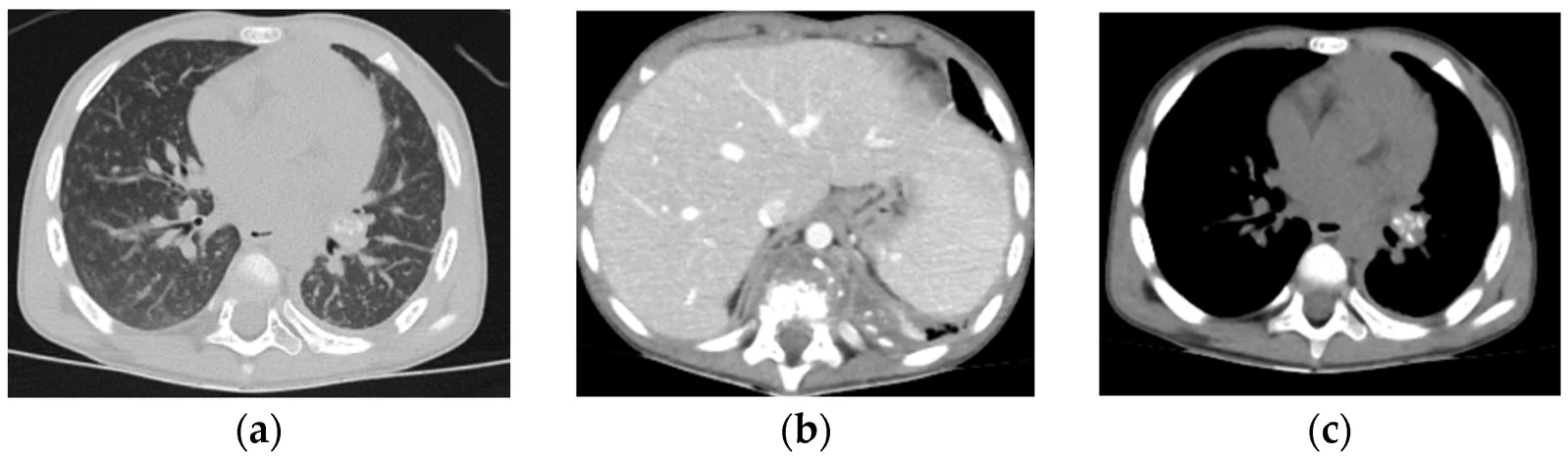
Contrast-enhanced chest and abdominal CT: (**a**) diffuse micronodular pulmonary lesions; (**b**) multiple splenic and hepatic micronodular lesions; (**c**) enlarged, partially calcified mediastinal lymph nodes.

Malignancy (such as neuroblastoma, Ewing sarcoma, or lymphoma) was initially suspected due to the combination of a firm, rapidly enlarging dorsal mass, osteolytic destruction of the D10 vertebra, a contiguous subpleural mass, and a frontal brain lesion in a young child presenting with weight loss and fever. Given the suspicion of an underlying neoplastic process, cerebrospinal fluid (CSF) analysis was performed. CSF chemical and physical parameters showed a leukocyte count of 28 cells/µL, with a predominance of mononuclear cells (96.4%) and 3.6% polymorphonuclear cells. Cytological examination documented inflammatory cells, including histiocytes, granulocytes, and lymphocytes, with no evidence of neoplastic cells. Bacterial cultures were negative. Based on these findings, TB was suspected. The Quantiferon-TB-Gold test was therefore performed and yielded a positive result. Household contact tracing was performed immediately. Screening of family members identified active pulmonary tuberculosis in the father, who represented the primary source of infection and was promptly initiated on anti-TB therapy. In addition, the patient’s three older siblings were screened: one sibling was negative, whereas two were diagnosed with pulmonary tuberculosis and received a 4-month shortened anti-TB treatment. To further characterize the disease, contrast-enhanced chest and abdominal computed tomography (CT) was performed (Figure 3). The examination revealed diffuse pulmonary micronodular changes suggestive of miliary pulmonary TB, a left subpleural collection extending into the paravertebral region from D9 to D11, and infiltration of the D10 vertebral body with osteolytic changes. Multiple enlarged, partially calcified lymph nodes were also detected in the hilar, mediastinal, and abdominal regions.

**Figure 4:**
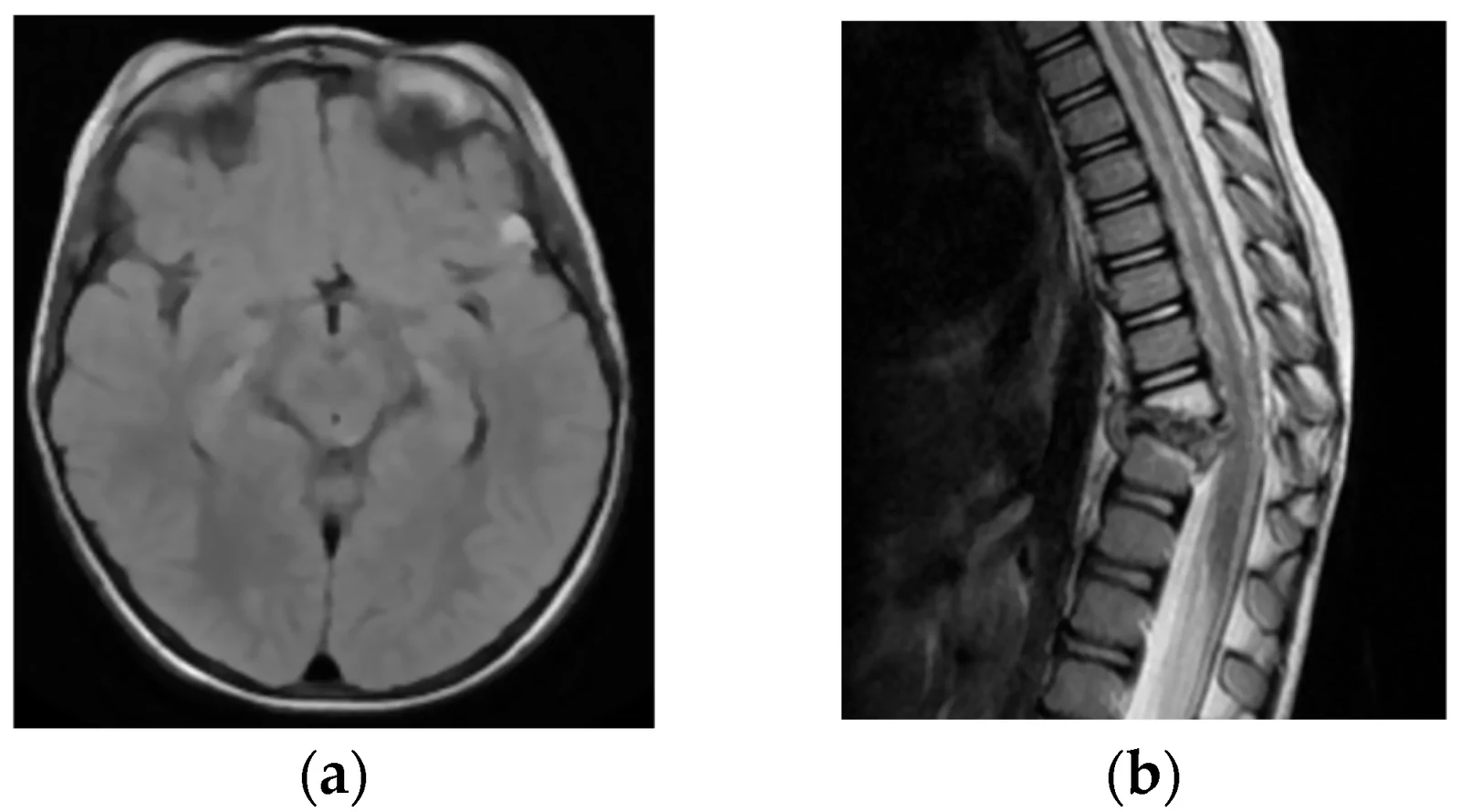
One-month follow-up MRI of the brain and spine: (**a**) reduction in the size of the left frontal tuberculoma; (**b**) marked decrease in the paravertebral abscess.

For microbiological diagnosis, the patient underwent bronchoscopy, and molecular testing (PCR) performed on the bronchoaspirate sample was positive for MTB. Therefore, three months after symptom onset, a diagnosis of Pott’s disease associated with disseminated miliary tuberculosis was established, with pulmonary, cerebral, cerebellar, hepatic, and splenic involvement. Antituberculosis therapy was started according to the 2025 WHO guidelines, with isoniazid (10 mg/kg/day), rifampicin (15 mg/kg/day), pyrazinamide (35 mg/kg/day), and ethambutol (20 mg/kg/day). The planned total duration of anti-TB treatment is 12 months (2 months of intensive HRZE regimen followed by 10 months of HR continuation phase), given the central nervous system and osteoarticular involvement. The patient showed good clinical tolerance throughout hospitalization, with no evidence of drug-related toxicity on serial laboratory monitoring. The patient presented with brain and cerebellar tuberculomas on neuroimaging, but CSF chemistry showed mononuclear pleocytosis without classic marked hypoglycorrhachia/hyperproteinorrhachia. Neither molecular testing (PCR) nor mycobacterial culture for MTB was performed on the CSF at the referring hospital. Therefore, according to the Marais consensus criteria for TBM, the diagnosis is classified as “possible tuberculous meningitis/central nervous system tuberculosis” (manifested primarily as multiple intracranial tuberculomas with associated mild CSF pleocytosis) [7]. Given the involvement of the central nervous system (CNS), adjunctive corticosteroid therapy with dexamethasone (0.6 mg/kg/day) was administered for four weeks, followed by a gradual tapering regimen. Based on neurosurgical recommendations, a custom-made rigid brace was fitted, allowing the patient to ambulate independently without evidence of motor deficits. In parallel, given the severe, disseminated presentation, investigations were performed to rule out underlying primary or secondary immunodeficiencies. Serum immunoglobulin levels and lymphocyte subset analyses were within normal limits, and HIV serology was negative. Exome sequencing was performed to screen for Mendelian Susceptibility to Mycobacterial Disease (MSMD). A heterozygous variant in the IL12RB1 gene was identified. According to the American College of Medical Genetics and Genomics (ACMG), the variant is classified as a class 3 variant, namely a variant of uncertain significance (VUS), indicating that the available evidence is insufficient to establish whether it is pathogenic or benign. Therefore, the identification of this heterozygous VUS alone is insufficient to establish a diagnosis of MSMD. Functional immunological studies would be required to assess the biological effect of the variant and determine whether it has any pathogenic significance. One month after the initiation of therapy, follow-up MRI demonstrated clinical and radiological improvement, with a reduction in the size of the left frontal tuberculoma and a marked decrease in the paravertebral abscess, without evidence of compressive myelopathy (Figure 4).

**Figure 5:**
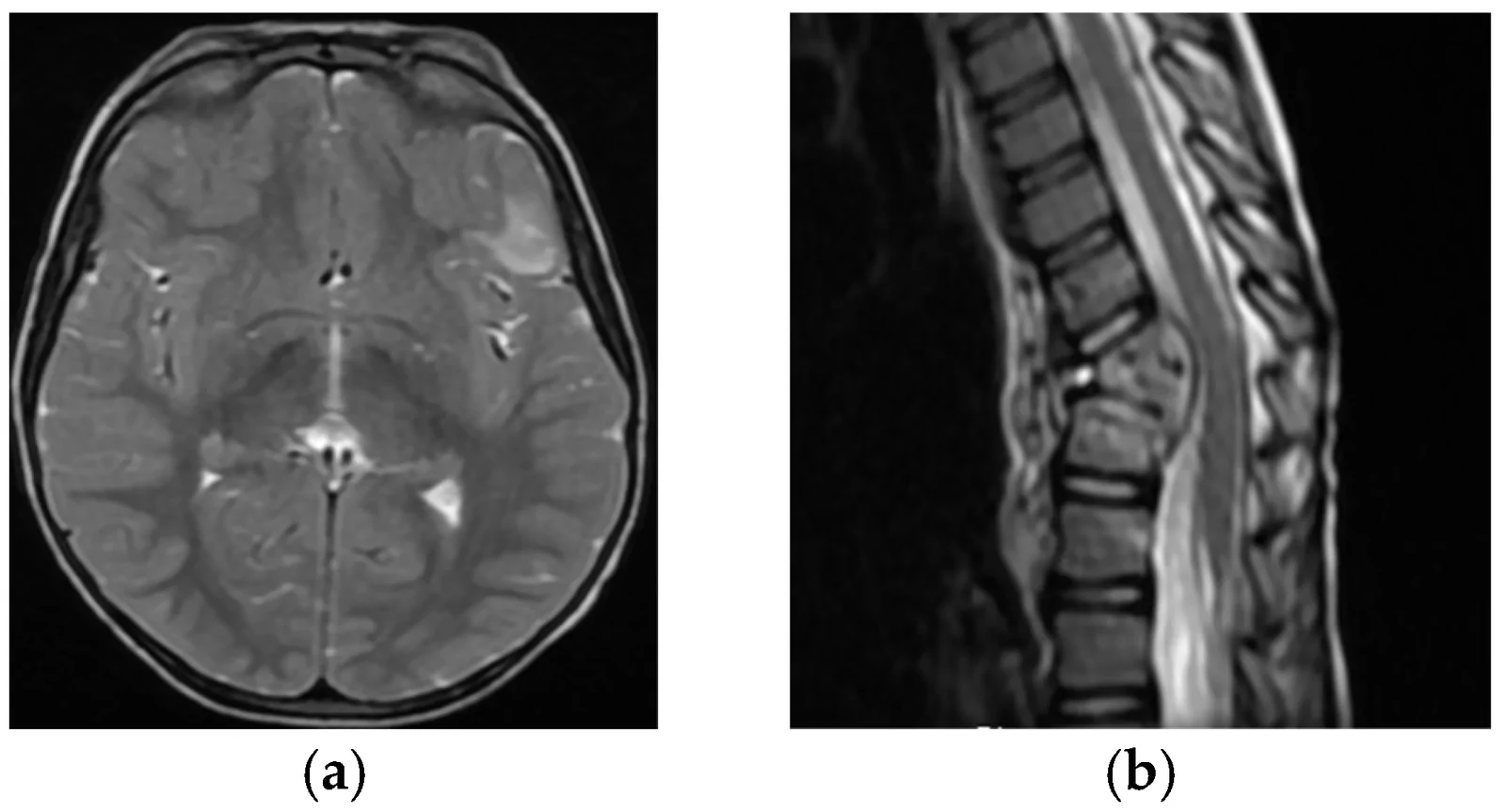
Three-month follow-up MRI of the brain and spine: (**a**) increased size of the left frontal tuberculoma; (**b**) enlargement of the paravertebral abscess, with involvement of the D11 vertebral body and initial involvement of D12, increased wedge-shaped deformity of D10, and progression of dorsal kyphosis.

After two months of hospitalization, the patient was discharged with instructions to continue brace use and ongoing treatment with isoniazid and rifampicin. MRI performed one month after discharge (approximately 3 months after the initiation of antituberculosis therapy) showed radiological worsening, with enlargement of both the paravertebral abscess and cerebral tuberculoma, as well as progression of dorsal kyphosis, compared with the one-month follow-up MRI (Figure 5).

Clinical and radiological worsening occurring at 3 months was attributed to immune reconstitution inflammatory syndrome (IRIS), more appropriately defined as a paradoxical reaction (PR) in HIV-negative patients. Before confirming PR, we systematically excluded alternative causes of lack of improvement. Treatment non-adherence was ruled out through daily directly observed administration during hospitalization and verified parental adherence post-discharge; drug resistance was excluded as gastric aspirate isolates demonstrated full susceptibility to all first-line anti-TB drugs. Therefore, corticosteroid therapy was reintroduced for approximately one month, resulting in clinical and radiological improvement at subsequent follow-up evaluations. At 10 months of clinical and neuroradiological follow-up, the patient is clinically and radiologically improved, with full, independent ambulation and no motor or sensory deficits. Neuroradiologically, marked reduction in paraspinal abscesses and brain tuberculomas was achieved, with a stable residual mild dorsal kyphotic deformity being managed conservatively with gradual brace weaning. Antituberculosis treatment with isoniazid and rifampicin is ongoing and is planned to be completed after 12 months of treatment.

## 3. Materials and Methods

We performed an exploratory literature review of published pediatric case reports on tuberculous spondylodiscitis using the PubMed and Scopus databases (January 2000 to August 2025). Given the exclusive inclusion of individual case reports, formal systematic review registration and Risk of Bias scoring were not applicable. The PubMed search string, finalized on 4 August 2025, was as follows: (((“discitis”[Tiab] OR “spondylodiscitis”[Tiab] OR “osteomyelitis”[Tiab] OR “bone infection”[Tiab] OR “vertebral infection”[Tiab] OR “skeletal”[Tiab] OR “bone”[Tiab] OR “spinal infection”[Tiab] OR “spinal”[Tiab] OR “vertebral”[Tiab] OR “spondylitis”[Tiab]) AND (“tuberculosis”[Tiab] OR “tuberculoses”[Tiab] OR “tuberculous”[Tiab] OR “TB”[Tiab] OR “TBC”[Tiab] OR “*Mycobacterium tuberculosis*”[Tiab])) OR (“Pott’s disease”[Tiab])) AND (“child”[Tiab] OR “children”[Tiab] OR “pediatric”[Tiab] OR “paediatric”[Tiab] OR “infant”[Tiab] OR “infants”[Tiab] OR “neonate”[Tiab] OR “neonates”[Tiab] OR “newborn”[Tiab] OR “adolescent”[Tiab]). The same conceptual search strategy was adapted to the syntax of the Scopus database. Retrieved records were initially screened by title and abstract, followed by full-text assessment of potentially eligible studies. Title and abstract screening was independently performed by two of the five authors, with each record assessed by two different reviewers. In cases of disagreement regarding eligibility, a third author reviewed the record and resolved the discrepancy. Full-text assessment and subsequent data extraction were performed independently by the individual authors. No automation tools were used during the screening, study selection, or data extraction processes. Filters were applied to focus the search on English-language articles published after January 2000. Case reports involving patients aged 0–17 years diagnosed with tuberculous spondylodiscitis were included. Epidemiological data, clinical manifestations, diagnostic methods, treatment, and outcome were collected. However, papers that did not report information on all these aspects were also included, clearly specifying when data were missing and what type of information was unavailable. Studies were excluded if they met any of the following criteria: publication before 2000; lack of an English-language full text; papers without patients’ data; reports involving only adult patients. The formal risk of bias assessment was not undertaken because of the exploratory descriptive design. Data were extracted using Microsoft Excel, version 16.112 (Microsoft Corporation, Redmond, WA, USA). Due to the inherent heterogeneity of clinical data from different case reports, the lack of standardization in data presentation, and the relatively small sample size, inferential statistical analyses were not performed. All results are summarized and presented descriptively.

## 4. Results

**Figure 6:**
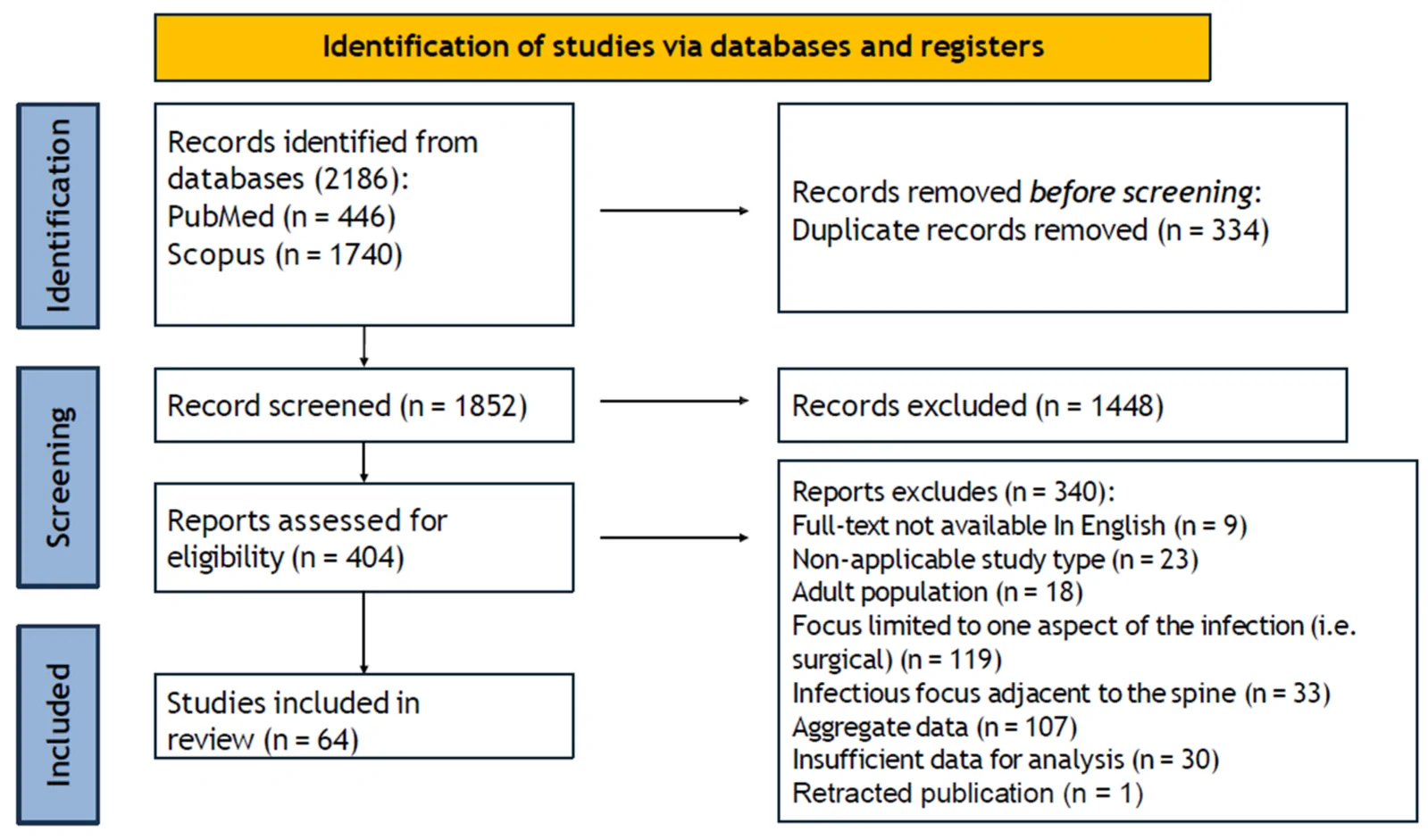
Flowchart describing the process of identifying and selecting studies.

The search yielded 2186 results, of which 446 were identified from the PubMed database and 1740 from Scopus. Of these, 334 were excluded because they were duplicates and 1448 because they were not relevant to TB spondylodiscitis. 404 results were then screened based on full-text analysis. Of these, 339 were excluded based on predefined criteria, as illustrated in Figure 6. 65 results, all case reports, were selected for review. One case report, which had been previously included in the review, was subsequently removed because the corresponding manuscript had been retracted. Therefore, the 64 papers report a total of 64 cases of TB spondylodiscitis in pediatric age [8,9,10,11,12,13,14,15,16,17,18,19,20,21,22,23,24,25,26,27,28,29,30,31,32,33,34,35,36,37,38,39,40,41,42,43,44,45,46,47,48,49,50,51,52,53,54,55,56,57,58,59,60,61,62,63,64,65,66,67,68,69,70,71].

Relevant data for each study, such as epidemiological and imaging characteristics, treatment, and outcomes, are summarized in Table 1. A more detailed overview of all collected data is provided in Supplementary Materials (Table S1).

### 4.1. Epidemiology

The studies included in this analysis spanned 31 distinct countries and involved a total of 64 patients. The origin of the patients did not always correspond to the countries in which the studies were conducted. Geographically, 62.5% (40/64) of cases originated from Asia, 14.1% (9/64) from Africa, 12,5% (8/64) from Europe, and 10.9% (7/64) from the Americas. Temporal distribution showed a steady publication rate between 2000 and 2012 (average 2 cases/year), increasing between 2013 and 2025 (average 3.5 cases/year). Of the entire cohort of cases included in this review, a significant proportion of 81.3% came from countries classified as having a high or moderate TB burden, according to the classification reported in the latest World Health Organization global list [72] (based on 2021 data and subject to revision in 2025). India was the most represented country (14 cases). Of the 64 cases, 34 were male (53.1%). The median age was 10 years (range 0.7–17 years).

### 4.2. Immunological Status

Of the 23 children with known vaccination status, 19 were BCG vaccinated. Two cases had comorbidities: one with type 1 diabetes mellitus and type 3 congenital lamellar ichthyosis, and the other with epilepsy. None were known to be HIV-positive, although only 32 patients were tested. Only one case, a BCG-vaccinated girl with cervical spondylitis and isolated *M. bovis*, was studied for MSMD; the exome was negative for specific mutations.

### 4.3. Clinical Manifestations

The mean duration of symptoms before diagnosis was 141.4 days. The most significant signs and symptoms, along with their frequencies, were collected in Table 2 using available data from 64 cases. Back pain was the most prevalent symptom (79.6%), followed by difficulty walking (68.4%) and constitutional symptoms, such as fever (55%) and weight loss (55.8%).

Thoracic involvement was the most frequent spinal localization (60.3%), followed by the cervical spine (31.7%), lumbar spine (30.2%), and sacral spine (6.3%). Osteolysis involved two or more vertebrae in most cases (29%). Paravertebral abscesses were described in 41.2% of cases. Pulmonary involvement was present in 46.4%. Central nervous system involvement in 38.2% of cases, and diffuse miliary TB in 27.8%. Subgroup stratification revealed notable differences: patients with cervical spine involvement had a higher incidence of retropharyngeal abscess (15% vs. 5.3% in thoracic cases) and airway/swallowing dysfunction. Patients managed in non-endemic countries experienced a longer median diagnostic delay compared to those in endemic areas (4 months vs. 3 months).

### 4.4. Diagnosis

In 44 of 57 cases (77.2%) for which microbiological testing information was available, MTB was confirmed by tests such as PCR, Ziehl-Neelsen (ZN) staining for acid-alcohol-fast bacilli, or culture. These tests were performed on various types of biological specimens, namely: sputum, gastric aspirate or lavage, paravertebral abscess drainage fluid, or bone biopsy. In 5 patients (7.8%), the histological findings reported were suggestive of MTB infection. Histological examination was performed on biopsy or surgical specimens. The diagnostic yield of the different microbiological methods is summarized in Table 3. In 13 cases (20.3%), the diagnosis was made based on epidemiological, clinical, radiological, and screening test criteria. Mantoux test results were reported in 36 patients (56.3%), of whom 30 were positive (83.3%). Of these, 12 patients (40%) had previously received the BCG vaccine. The Quantiferon-TB Gold test was performed in 12 patients (18.8%) and was positive in 11 cases (91.6%). Drug-resistant isolates were identified in 5 of the 44 patients who tested positive for MTB (11.4%), including 2 cases of multidrug-resistant tuberculosis (MDR-TB) (4.5%). These patients were all from highly endemic TB countries. Instrumental investigations documenting spinal involvement were performed in all patients, except for one child who died on arrival at the hospital. The following investigations were used: chest X-ray (81%); spinal MRI (60.3%); and chest CT (44.4%).

### 4.5. Treatment

All patients received hospital care and antituberculosis drugs, except for one child, whose TB spondylodiscitis was an autopsy finding. Second-line antituberculosis treatment was administered in five drug-resistant tuberculosis, including two cases of MDR-TB. Corticosteroid therapy was administered in seven cases. In four cases, corticosteroids were used in the setting of CNS involvement, while in one case they were administered for a retropharyngeal abscess associated with airway compression. In the remaining two cases, the specific indication for corticosteroid treatment could not be determined based on the clinical information reported in the respective papers. Thirty-six patients (58%) underwent surgery. Drainage of perivertebral abscesses was the most common surgical procedure performed. Debridement and laminectomy were performed to relieve spinal cord compression in patients with severe neurological manifestations. Screw stabilization was performed in patients with spinal instability.

### 4.6. Outcome

In 62 cases (96.9%), treatment resulted in clinical improvement (resolution of fever, pain, and constitutional symptoms). Neurological recovery (resolution of motor/sensory deficits) was achieved in 81.2% of those with initial deficits, while radiological recovery (abscess resolution and bone healing) was observed, with 14.5% retaining residual kyphotic deformity. Of these 62 recovered patients, 14 (22.6%) developed long-term sequelae, including 5 cases (8.1%) of lower limb paraparesis, with permanent loss of ambulation in one case, and 9 patients (14.5%) with persistent kyphotic deformity. Two patients (3.1%) with cervical spondylitis, both 13 years old, died: an Indian girl died on arrival at the hospital with a postmortem diagnosis of asphyxia due to a retropharyngeal abscess of TB origin; while the other patient, a Nigerian, died in the hospital while undergoing empirical therapy for TB, while awaiting drainage of a retropharyngeal abscess, from which fluid collected during the autopsy tested positive for MTB.

## 5. Discussion

Our analysis revealed that pediatric TB spondylodiscitis is most frequently reported in children around 10 years of age, with cases ranging from 8 months to 17 years. Disseminated miliary TB was an uncommon finding, reported in only 10 patients (15.7%) among the published cases. Most pediatric cases of TB spondylodiscitis reported in the literature originate from high TB incidence countries, with a marked predominance of South Asian regions, particularly India [73]. In low-endemic countries, this condition is more frequently observed among children born to migrant parents from countries with a high TB burden [73]. These children, often referred to as “second-generation children,” represent a distinct epidemiological group. Although they have a lower incidence of TB disease compared with foreign-born children, their risk remains approximately 2.2-fold higher than that of children born in non-endemic countries (10.2 vs. 4.6 per 100,000) [74,75]. Although routine BCG vaccination is not recommended for the general pediatric population in Italy, specific indications apply to children at increased risk of TB, including those with a parent or grandparent born in a country with a high incidence of TB [76]. Our patient was born in Italy to Romanian parents and therefore met the criteria for BCG vaccination. However, he had not received BCG vaccination. Our patient represents an unusual presentation due to his very young age (<2 years) and the miliary TB, with simultaneous osteoarticular, spinal, and central nervous system involvement. Miliary TB results from massive lymphohematogenous dissemination of MTB from a primary pulmonary focus, with bacterial spread through the bloodstream to multiple organs due to inadequate immune control of the primary infection [77]. In children, systemic dissemination occurs more frequently during early post-primary TB and is characterized by an acute onset and rapid progression [77]. Vertebral involvement in disseminated TB typically begins in the anterior portion of the vertebral body adjacent to the intervertebral disc, leading to progressive bone destruction and, in some cases, the formation of paravertebral abscesses [78]. The clinical presentation and diagnostic course of pediatric spinal TB may differ from that observed in adults. In children, symptoms may be nonspecific and neurological manifestations may be subtle or absent initially, whereas the growing spine is particularly susceptible to progressive structural deformity. Compared to adult TB spondylodiscitis, which typically presents as localized lumbar involvement with lower rates of acute systemic spread, pediatric cases—especially in infants—show faster progression and higher rates of multi-segmental destruction. This difference may be related to the anatomical and immunological characteristics of the growing spine, including the rich vascular supply of the vertebral growth regions and the immaturity of the immune response, which also increase the long-term risk of progressive spinal deformities compared to adults [79]. Due to the rarity of TB spondylodiscitis in low-endemic countries, including our own, diagnosis is frequently delayed, particularly in children. Back pain is the most common presenting symptom (79.6%), followed by difficulty walking (68.4%) and systemic manifestations such as fever (55%), weight loss (55.8%), and hyporexia (47.7%). In the presence of these clinical features, the initial diagnostic approach often focuses on rheumatological or neoplastic conditions, while TB is considered only after these more common aetiologies have been excluded [80]. Neurological manifestations, when present, usually are subacute and may further contribute to delays in diagnosis [80]. This diagnostic delay is clinically relevant because progressive vertebral destruction may occur before the diagnosis is established, increasing the risk of deformity and neurological complications. Although the identification of MTB represents the diagnostic gold standard, it is not always achievable in children. Our literature review showed that, among the 57 cases in which microbiological investigations were performed, MTB was identified by PCR, Ziehl–Neelsen (ZN) staining, or culture in 77.2% of patients. In the remaining cases, diagnosis was established based on clinical, radiological, and epidemiological criteria, associated with immunological tests such as Mantoux tuberculin skin test and Quantiferon assay. To limit this diagnostic delay, spinal MRI represents the gold standard for diagnosis of pediatric spinal TB, allowing early detection of subtle vertebral marrow edema, neurological involvement, and soft-tissue abscesses, often before structural vertebral collapse becomes evident on plain radiography [81]. MRI is therefore particularly valuable in children, in whom early recognition of spinal and neurological involvement may help avoid irreversible complications. In addition, spinal MRI is essential for identifying potential indications for surgical intervention. The high proportion of surgical interventions observed in our review (58%) can be explained by the severity of the clinical presentations reported, particularly the presence of spinal cord compression and paravertebral abscesses requiring drainage. This finding suggests that surgery was primarily reserved for patients with advanced or complicated spinal disease. Drainage of paravertebral abscesses was the most frequently performed procedure, followed by debridement and laminectomy, whereas spinal stabilization with plates and screws was reserved for children with vertebral instability. These indications are consistent with the existing evidence, according to which surgery is generally considered in the presence of neurological deficits, spinal instability, severe or progressive kyphosis, large or persistent abscesses, or failure to respond adequately to anti-TB treatment [82]. In our case, the absence of an indication for urgent surgical decompression or stabilization allowed conservative management with a rigid brace, combined with anti-TB and corticosteroid therapy, resulting in clinical and radiological improvement and avoiding a surgical approach. Nevertheless, the persistence of dorsal kyphotic deformity during follow-up highlights the importance of prolonged clinical and radiological surveillance in children, given the potential for deformity progression during growth. Underlying immunodeficiency should always be considered and investigated in children presenting with particularly severe or disseminated forms of TB. In our case, clinical exome analysis documented a heterozygous mutation in innate immunity associated with MSMD [83,84]. MSMD is a group of rare primary immunodeficiencies, characterized by a selective impairment of cell-mediated immunity [85]. These defects compromise the host response to MTB infections due to altered activation of the interleukin-12/interferon-γ (IL-12/IFN-γ) pathway, which plays a crucial role in macrophage and natural killer (NK) cell activation and in the development of an effective adaptive immune response against MTB [86]. Genetic analysis identified a heterozygous IL12RB1 variant. According to ACMG guidelines, the variant is classified as a class 3 variant (VUS), indicating that the available evidence is insufficient to establish whether it is pathogenic or benign [87]. Therefore, the identification of this heterozygous VUS alone is insufficient to establish a diagnosis of MSMD or to attribute the patient’s severe clinical presentation to the variant. Functional immunological studies would be required to assess the biological effect of the variant and determine whether it has any pathogenic significance. Thus, this finding should be interpreted cautiously and considered only as a possible contributing factor rather than a demonstrated cause of the patient’s susceptibility to mycobacterial infection. Drug-resistant tuberculosis (DR-TB) represents a growing global concern that should be considered in pediatric patients, particularly those with epidemiological links to high-burden regions. DR-TB may significantly complicate management of spinal TB, requiring second-line treatment regimens and potentially affecting clinical and neurological outcomes. Although our review did not identify drug resistance as a major feature of the included pediatric cases, susceptibility testing remains essential whenever MTB is microbiologically confirmed, particularly in severe or disseminated disease [88]. In our patient, the isolate obtained from gastric aspirate was a fully drug-sensitive MTB, supporting the use of standard first-line therapy and helping to exclude drug resistance as an explanation for the subsequent clinical and radiological deterioration. Regarding the therapeutic approach, according to the 2025 WHO guidelines, the recommended first-line treatment for pediatric osteoarticular TB consists of a two-month intensive phase with isoniazid, rifampicin, pyrazinamide, and ethambutol (HRZE), followed by a 10-month continuation phase with isoniazid and rifampicin (HR) [89]. In cases of concomitant CNS involvement, as observed in our patient, adjunctive corticosteroid therapy is recommended, consisting of dexamethasone or prednisone for four weeks followed by progressive tapering for a further 4 weeks [90]. This therapeutic regimen was also followed in our patient, with clinical benefit. One month after corticosteroid discontinuation, and approximately 90 days after the initiation of antituberculosis therapy, our patient developed clinical and radiological worsening, with progression of the vertebral necrotic-colliquative lesion, compatible with the diagnosis of IRIS. In HIV-negative patients, IRIS is more appropriately defined as a PR [90]. It consists of clinical and/or radiological worsening occurring during effective antituberculosis therapy, in the absence of alternative documented causes [90]. In our patient, the diagnostic criteria for PR were fulfilled: the clinical and radiological deterioration occurred after the initiation of appropriate anti-TB therapy, while treatment failure due to drug resistance and poor adherence were excluded. In fact, MTB isolated from gastric aspirate was susceptible to all tested anti-TB drugs, and adherence to treatment at home was reported to be regular. PR is linked to the restoration of the host immune response against MTB, which does not necessarily correlate with an increase in CD4 cell count, resulting in the exacerbation of inflammatory and necrotic processes, worsening of pre-existing lesions, and/or the development of new infectious foci [91]. In our patient, the CD4+ lymphocyte count obtained during the PR was within the normal range; however, a baseline CD4+ count at the time of tuberculosis diagnosis was not available. IRIS is rare in the pediatric population and has been reported more frequently in younger children with severe neurological involvement and disseminated TB [91]. No cases of IRIS were reported among the cases included in our analysis. The management of IRIS is primarily based on corticosteroid therapy, although pediatric evidence remains limited and insufficient to define the optimal treatment strategy [91]. In our patient, reintroduction of corticosteroids resulted in clinical and radiological improvement. Regarding clinical outcomes, data from our review showed that, despite a high rate of recovery, TB spondylodiscitis may result in long-term sequelae, particularly kyphotic spinal deformities, which represent a significant finding in the growing pediatric population. In our case as well, at the tenth month of clinical and neuroradiological follow-up, a dorsal kyphotic deformity persists despite a favourable response to antituberculosis therapy. Treatment is ongoing and will be continued for a duration of 12 months. This review has several strengths and limitations. A major strength is the focus on the rare presentation of pediatric TB spondylodiscitis, including clinical presentation, diagnostic findings, treatment, complications, and outcomes. The systematic search of two major biomedical databases and the independent screening process also strengthen the identification and selection of relevant cases. However, the evidence available in the literature is limited by the rarity of the condition and by the predominance of case reports, which are subject to publication and selection bias and do not allow reliable estimates of incidence or comparative assessment of treatment strategies. Furthermore, the information reported across cases was heterogeneous and incomplete, with some publications lacking data on specific clinical, microbiological, therapeutic, or follow-up aspects. Given the exploratory and descriptive nature of this review, a formal risk of bias assessment was not performed. The absence of a control group and the retrospective nature of the available evidence also limit the possibility of drawing causal conclusions. Finally, our findings should therefore be interpreted as a descriptive synthesis of the available literature rather than as evidence of comparative effectiveness of specific diagnostic or therapeutic approaches.

## 6. Conclusions

Although uncommon in low-endemic countries, TB spondylodiscitis should not be overlooked in children. Early diagnosis through spinal MRI, combined with microbiological confirmation, and prompt treatment of Pott’s disease are critical to improving outcomes and preventing severe complications, including neurological sequelae, progressive spinal deformities, and potentially life-threatening outcomes, particularly in the growing spine.

## Figures and Tables

**Table 1:** Characteristics and outcomes of reported cases.

Study Findings	*N* (%)
Age, years (mean)	8.58 (range 0.7–17 years)
Sex:	
Male	34/64 (53.1%)
Female	30/64 (46.9%)
Country of birth:	
Low-endemic countries	12/64 (18.7%)
High-endemic countries	52/64 (81.3%)
Mean duration of symptoms before diagnosis (days)	141.4 days
Most prevalent signs and symptoms:	
Back pain	47/59 (79.7%)
Difficulty walking	39/57 (68.4%)
Fever	28/51 (55%)
Weight loss	24/43 (55.8%)
Diagnostic imaging:	
XR	51/63 (81%)
MRI	38/63 (60.3%)
CT	28/63 (44.4%)
Vertebral involvement:	
Thoracic	38/63 (60.3%)
Lumbar	19/63 (30.2%)
Cervical	20/63 (31.7%)
Sacral	4/63 (6.3%)
Treatment:	
Pharmacological treatment	63/64 (98.4%)
Surgical treatment	36/62 (58%)
Clinical outcome:	
Recovery	62/64 (96.9%)
Sequelae	14/62 (22.6%)
Death	2/64 (3.1%)

**Table 2:** Main signs and symptoms of reported cases.

Signs and Symptoms	*N* (%)
Back pain	47 (79.6%)
Difficulty walking	39 (68.4%)
Fever	28 (55%)
Weight loss	24 (55.8%)
Hyporexia	21 (47.7%)
Paresthesia of lower limbs	20 (43.5%)
Lymphadenopathy	13 (34.2%)
Motor deficit of upper limbs	12 (25.5%)
Urinary incontinence	5 (14.3%)
Dyspnea	4 (9.3%)
Tachypnea	4 (9.1%)
Fecal incontinence	3 (8.6%)
Cyanosis	2 (5.1%)

**Table 3:** Diagnostic findings of PCR, culture, and ZN in the reported cases.

Diagnostic Method	Positive Cases/Tested	Diagnostic Yield (%)
PCR (GeneXpert/MTB PCR)	23/29	79.3%
Mycobacterial Culture	10/13	76.9%
Acid-Fast Stain (Ziehl-Neelsen)	13/23	56.5%

## Data Availability

No new data were created or analyzed in this study. Data sharing is not applicable to this article.

## Supplementary Materials

The following supporting information can be downloaded at: https://www.mdpi.com/article/10.3390/pathogens15090919/s1, Table S1. Overview of relevant data collected from each study. M “male”; F “female”; C “cervical”; T “thoracic”; L “lumbar”; S “sacral”; NP “not performed”; NR “not reported”; PCR “polymerase chain reaction”; ZN “Ziehl-Neelsen staining”; H “isoniazid”; “R” rifampicin; “Z” pyrazinamide; “E” ethambutol”. References [8,9,10,11,12,13,14,15,16,17,18,19,20,21,22,23,24,25,26,27,28,29,30,31,32,33,34,35,36,37,38,39,40,41,42,43,44,45,46,47,48,49,50,51,52,53,54,55,56,57,58,59,60,61,62,63,64,65,66,67,68,69,70,71] are cited in the Supplementary Materials.

## Abbreviations

TB	Tuberculosis
MTB	*Mycobacterium tuberculosis*
MRI	Magnetic resonance imaging
CSF	Cerebrospinal fluid
CNS	Central nervous system
ZN	Ziehl-Neelsen
MSMD	Mendelian Susceptibility to Mycobacterial Disease
NK	Natural killer
IRIS	Immune Reconstitution Inflammatory Syndrome
PR	Paradoxical reaction
CT	computed tomography
VUS	Variant of uncertain significance

## References

[1] Moroni M. (2020). Manuale di Malattie Infettive.

[2] World Health Organization (2025). Global Tuberculosis Report 2025.

[3] Eisen S., Honywood L., Shingadia D., Novelli V. (2012). Spinal tuberculosis in children. Arch. Dis. Child..

[4] Johansen I.S., Nielsen S.L., Hove M., Kehrer M., Shakar S., Wøyen A.V., Andersen P.H., Bjerrum S., Wejse C., Andersen Å.B. (2015). Characteristics and clinical outcome of bone and joint tuberculosis from 1994 to 2011: A retrospective register-based study in Denmark. Clin. Infect. Dis..

[5] Kamara E., Mehta S., Brust J.C., Jain A.K. (2012). Effect of delayed diagnosis on severity of Pott’s disease. Int. Orthop..

[6] Rajasekaran S. (2013). Natural history of Pott’s kyphosis. Eur. Spine J..

[7] Marais S., Thwaites G., Schoeman J.F., Török M.E., Misra U.K., Prasad K., Donald P.R., Wilkinson R.J., Marais B.J. (2010). Tuberculous meningitis: A uniform case definition for use in clinical research. Lancet Infect. Dis..

[8] Bozzola E., Bozzola M., Magistrelli A., Calcaterra V., Larizza D., Lancella L., Villani A. (2013). Paediatric tubercular spinal abscess involving the dorsal, lumbar and sacral regions and causing spinal cord compression. Infez. Med..

[9] Hugar B.S., Chandra Y.P., Babu P.R., Jayanth S.H., Vinay J. (2013). Fatal case of retropharyngeal abscess associated with Pott’s disease. J. Forensic Leg. Med..

[10] Idris S.K., Abdulkadir A.Y. (2010). Tuberculous retropharyngeal abscess with posterior mediastinal extension and quadriplegia in a 13-year-old Nigerian girl. Int. J. Pediatr. Otorhinolaryngol. Extra.

[11] Goh J.L.K., Chong C.Y., Lim S.Z.R., Lim K.B.L., Tan N.W.H. (2018). A case of tuberculosis spondylodiscitis with paraspinal abscess in a 2-year-old child. Glob. Pediatr. Health.

[12] Kapoor S.K., Tiwari A., Chaudhry A. (2007). An unusual case of craniovertebral junction tuberculosis in an infant. Spine.

[13] Diom E.S., Ndiaye C., Djafarou A.B., Ndiaye I.C., Faye P.M., Tall A., Ndiaye M., Diallo B.K., Diouf R., Diop E.M. (2011). A case of cervical Pott’s disease revealed by parapharyngeal abscess. Eur. Ann. Otorhinolaryngol. Head Neck Dis..

[14] Dagli C.E., Guler E., Bakan V., Atilla N., Koksal N. (2009). Miliary tuberculosis accompanying paravertebral tuberculosis abscess in an adolescent. J. Infect. Dev. Ctries..

[15] Mohta S., Kumar A., Singh N., Wig N. (2017). A case of tuberculous gumma: There is more to it than meets the eye. BMJ Case Rep..

[16] Aktürk H., Hançerli Törün S., Çalışkan B., Erol O.B., Gün F., Salman N., Somer A. (2015). A late diagnosis of Pott’s disease in an adolescent with psoas abscess. Marmara Med. J..

[17] Parent A.S., Tu A. (2024). Conservative management for pediatric craniocervical Pott’s disease: A case study and literature review. Childs Nerv. Syst..

[18] Chilundo J., Muhelo A., Ahivaldino Z., Zucula H., Macuácua S., Mussagi A.C., Pizzol D., Smith L., Maggioni G. (2023). Successful management, in a low-resource setting, of disseminated tuberculosis in a 3-year old boy: A case report. Pathogens.

[19] Boussetta R., Zairi M., Sami S.B., Lafrem R., Msakeni A., Saied W., Nessib N. (2020). Torticollis as a sign of spinal tuberculosis. Pan Afr. Med. J..

[20] Cheung J.P., Ho K.W., Lam Y.L., Shek T.W. (2012). Unusual presentations of osteoarticular tuberculosis in two paediatric patients. BMJ Case Rep..

[21] Yigit O., Erol M., Bostan Gayret O., Ustun I., Ulas S. (2016). Coexistence of a Ghon complex, Pott’s disease, and hip arthritis in a child. Iran. Red Crescent Med. J..

[22] Cassimos D., Tsalkidis A., Gardikis S., Lazopoulou N., Oikonomou A., Vaos G., Kambouri K., Verettas D., Theodoridou M., Chatzimichael A. (2009). Pott’s disease in a two-year-old girl. Minerva Pediatr..

[23] Khattry N., Thulkar S., Das A., Khan S.A., Bakhshi S. (2007). Spinal tuberculosis mimicking malignancy: Atypical imaging features. Indian J. Pediatr..

[24] Varo R., Bila R., Saavedra B., Sitoe A., Uamusse A., Ribó-Aristizabal J.L., García-Basteiro A.L. (2019). Paraplegia and spinal deformity in a Mozambican child with Pott’s disease and tuberculous scrofula. Lancet.

[25] Verma R., Junewar V., Garg R.K., Malhotra H.S. (2012). A rare case of basilar impression. BMJ Case Rep..

[26] Taccari F., Baldin G., Emiliozzi A., Campana L., Tamburrini E., Leone E., Delogu G., Sali M., Cauda R., Leone A. (2019). Case report: Multifocal tubercular osteomyelitis of the spine and bilateral dactylitis. Am. J. Trop. Med. Hyg..

[27] Singh S.N., Bhatt T.C., Kumar S., Chauhan V., Pandey A. (2016). A case of cervical spine tuberculosis in an infant. J. Clin. Diagn. Res..

[28] Ateş G., Özmen C.A., Yıldız T., Kışın B., Akyıldız L. (2011). Multiple metastatic tuberculosis abscesses and Pott’s disease in an immunocompetent patient: Case report. Turk. Klin. J. Med. Sci..

[29] Leos-Leija A.K., Padilla-Medina J.R., Reyes-Fernández P.M. (2022). Vertebral destruction in an 11-month-old child with spinal tuberculosis: A case report and review of literature. Ann. Pediatr. Surg..

[30] Albarrak M., Alodayani A., Al Otaibi N., Albrikeet Y. (2021). Pott’s disease with incidentally discovered multiple brain tuberculomas in a previously healthy 10-year-old girl. Case Rep. Infect. Dis..

[31] Shah S.S., Goregaonkar A.A., Goregaonkar A.B. (2017). Extensively drug-resistant tuberculosis of the lumbar spine in a six-year-old child: A case report. J. Orthop. Case Rep..

[32] Wang C., Luan Y., Liu S., Zhao M., Zhang H., Li W., Li Z., Hu X., Peng L. (2020). Multifocal tuberculosis simulating a cancer: A case report. BMC Infect. Dis..

[33] Song D.I., Sohn S.Y., Kim Y.K., Eun S.H., Rhie Y.J., Jang G.Y., Woo C.W., Choi B.M., Lee J.H., Je B.K. (2010). A childhood case of spinal tuberculosis misdiagnosed as muscular dystrophy. Korean J. Pediatr..

[34] Nakamura Y., Fumimoto R., Goto R., Moriuchi T., Niiya R., Torii Y., Katsuta T. (2025). A case of pediatric spinal tuberculosis contracted in a high-endemic country and developed in a low-endemic country. Pediatr. Int..

[35] Das A., Gupta V., Anupurba S. (2021). Rifampicin-resistant disseminated tuberculosis in an immunocompetent adolescent male presenting with retropharyngeal abscess and spinal involvement. J. Lab. Physicians.

[36] Tian Y., Shen X., Wang X., Zhou X., Yuan W. (2013). Tuberculosis of the lower cervical spine (C5–C6) in a 24-month-old infant. Spine J..

[37] Siwele B.A., Makhado N.A., Mariba M.T. (2019). Late diagnosis of multidrug-resistant tuberculosis in a child at Dr George Mukhari Academic Hospital, Ga-Rankuwa, South Africa: A case report. Afr. J. Lab. Med..

[38] Reilly C., Steinbok P., Pike J. (2005). Cervical intramedullary tuberculoma and tuberculous kyphosis in a 23-month-old child: Case report. Can. J. Surg..

[39] Goh J.H., Fazir M., Zainal-Abidin N.A., Amir D., Singh M. (2016). Acute paraplegia in a toddler: A diagnostic journey compounding the challenge in management: A case report. Malays. Orthop. J..

[40] Jasińska N., Bąk J., Andruszkiewicz K., Zawadzka M., Limanówka M. (2025). A rare case of Pott’s disease in a 10-year-old female patient of Indian origin. Przegl Epidemiol..

[41] Imakiire R., Nishikawa T., Tominaga H., Tawaratsumida H., Imuta N., Koriyama T., Nishi J., Taniguchi N., Kawano Y. (2020). Bacillus Calmette-Guérin-associated cervical spondylitis in a 3-year-old immunocompetent girl. Pediatr. Infect. Dis. J..

[42] Masavkar S., Shanbag P., Inamdar P. (2012). Pott’s spine with bilateral psoas abscesses. Case Rep. Orthop..

[43] Alawad A.A., Khalifa A.F. (2013). Cervical Pott’s disease presenting as a retropharyngeal abscess: Controlled by aspiration and anti-tuberculous chemotherapy. Sudan. Med. J..

[44] Emel E., Güzey F.K., Güzey D., Bas N.S., Sel B., Alatas I. (2006). Non-contiguous multifocal spinal tuberculosis involving cervical, thoracic, lumbar and sacral segments: A case report. Eur. Spine J..

[45] Cucuzza M.E., Mendola F., D’Ambra A., Smilari P., Greco F., Fiumara A., Praticò A.D. (2018). Pott disease in a 14-year-old girl affected by congenital lamellar ichthyosis type 3 and diabetes mellitus. J. Glob. Infect. Dis..

[46] Lee I.C., Quek Y.W., Tsao S.M., Chang I.C., Sheu J.N., Chen J.Y. (2010). Unusual spinal tuberculosis with cord compression in an infant. J. Child Neurol..

[47] Van Well G.T., Van der Mark L.B., Vermeulen R.J., Van Royen B.J., Wuisman P.I., Van Furth A.M. (2007). Spinal tuberculosis in a 14-year-old immigrant in the Netherlands. Eur. J. Pediatr..

[48] Giridharan P., Selladurai E., Balaji S., Pramila S.K., Arunagirinathan V., Shanmugam S., Hissar S., Natrajan M., Dooley K.E., Daniel B.D. (2020). Drug resistant TB spine in a two year old child: A case report. Indian J. Tuberc..

[49] Senanayake M.P., Karunaratne I. (2014). A child presenting with tuberculous spondylitis in a single third cervical vertebra: A case report. J. Med. Case Rep..

[50] Basnet P., Joshi A., Karki S., Thapa A.J., Poudel P., Sapkota A., Shrestha M., Pande S. (2024). Pott’s paraplegia in a 2 years female: A rare presentation at an early age. Case Rep. Infect. Dis..

[51] Anikoh S., Nwaneri U.D., John O., Orbih E., Sadoh A.E. (2024). Pott’s disease in a two year old: A consequence of failed contact tracing. Niger. J. Paediatr..

[52] Bayhan G.I., Tanir G., Gayretli Aydın Z.G., Yildiz Y.T. (2015). Miliary tuberculosis disease complicated by Pott’s abscess in an infant: Seven year follow-up. Lung India.

[53] Aouraghe H., Benchekroun S., Mahraoui C., ElHafidi N. (2023). Multifocal tuberculosis in children: A case of spinal tuberculosis. Int. J. Mycobacteriol..

[54] Haghighatkhah H., Jafroodi Y., Sanei Taheri M., Pourghorban R., Sadeghian Dehkordy A. (2015). Multifocal skeletal tuberculosis mimicking Langerhans cell histiocytosis in a child: A case report with a long-term follow-up. Iran. Red Crescent Med. J..

[55] Shukla S., Singh S., Puri V., Verma D., Jain L. (2011). Bilateral symmetrical facial swelling owing to tuberculous gummas. Ann. Trop. Paediatr..

[56] Papan C., Von Both U., Kappler M., Kammer B., Griese M., Huebner J. (2017). Pott’s disease: A major issue for an unaccompanied refugee minor. Thorax.

[57] Osguthorpe R.J., Parker S., Nyquist A.C. (2001). Case report: Two-year-old boy with a limp. Curr. Opin. Pediatr..

[58] Oberdorfer P., Kongthavonsakul K., Lochungvu H.P. (2012). A 3-year-old boy with kyphosis, back mass and weakness. BMJ Case Rep..

[59] Ochoa T.J., Rojas R., Gutierrez M., Porturas D. (2006). Severe airway obstruction in a child with Pott’s disease. Pediatr. Infect. Dis. J..

[60] Kiymaz N., Yilmaz N., Demir O. (2006). Spinal cord compression from spinal tuberculosis in a child. Pediatr. Neurosurg..

[61] Handryastuti S., Kaswandani N., Hendriarto A., Tobing S.D.A.L., Rafli A. (2024). Clinical manifestations and prognosis of tuberculous spondylitis in an adolescent with disseminated tuberculosis: A case report. Paediatr. Indones..

[62] Posner K.R., Mittal M.K. (2010). A teenage girl with a sternal mass: An unusual presentation of Pott disease. Pediatr. Emerg. Care.

[63] Locham K.K., Garg R., Singh M. (2001). Tuberculosis of lower cervical spine. Indian Pediatr..

[64] Avadhani A., Shetty A.P., Rajasekaran S. (2010). Isolated tuberculosis of the lumbar apophyseal joint. Spine J..

[65] Afzal A., Arshad M., Ashraf O. (2006). Psoas abscess secondary to Pott’s disease—An unusual presentation in a young child. J. Pak. Med. Assoc..

[66] Ferris B., Gonzalez M.D., Fox T., Kao C.M. (2020). Multifocal osteomyelitis in a child presenting with a mediastinal mass. Clin. Pediatr..

[67] Sutantoyo F.F., Sugianto P. (2022). A malignant disseminated tuberculosis: Concurrent intracranial tuberculosis with skipped multilevel spondylitis in a young immunocompetent patient. Rom. J. Neurol..

[68] Manoharan S.R., Leitao J., Emberton P., Quraishi N.A. (2013). A large tuberculosis abscess causing spinal cord compression of the cervico-thoracic region in a young child. Eur. Spine J..

[69] Tufan K., Dogulu F., Kardes O., Oztanir N., Baykaner M.K. (2005). Dorsolumbar junction spinal tuberculosis in an infant: Case report. J. Neurosurg..

[70] Garg A., Wadhera R., Gulati S.P., Kishore D., Singh J. (2009). Giant retropharyngeal abscess secondary to tubercular spondylitis. Indian J. Tuberc..

[71] Soeroso N.N., Pradana A., Lubis N., Soeroso L. (2018). Successful treatment of total paraplegic patient due to tuberculous spondylitis. Respirol. Case Rep..

[72] World Health Organization (2021). WHO Global Lists of High Burden Countries for Tuberculosis (TB), TB/HIV and Multidrug/Rifampicin-Resistant TB (MDR/RR-TB), 2021–2025.

[73] Gouveia C., Cabral M.F., Jordão P. (2022). Bone and joint tuberculosis in paediatrics: A 13-year retrospective study. J. Med. Microbiol..

[74] Marx F.M., Fiebig L., Hauer B. (2015). Higher rate of tuberculosis in second generation migrants compared to native residents in a metropolitan setting in Western Europe. PLoS ONE.

[75] Dara M., Dadu A., Kremer K. (2013). Epidemiology of tuberculosis in WHO European Region and public health response. Eur. Spine J..

[76] Ministero della Salute, Italia (2010). Aggiornamento Delle Raccomandazioni per le Attività di Controllo Della Tubercolosi: Gestione dei Contatti e Della Tubercolosi in Ambito Assistenziale.

[77] Sharma S.K., Mohan A., Sharma A. (2005). Miliary tuberculosis: New insights into an old disease. Lancet Infect. Dis..

[78] Lew D.P., Waldvogel F.A. (2004). Osteomyelitis. Lancet.

[79] Rajasekaran S., Shanmugasundaram T.K., Prabhakar R., Dheenadhayalan J., Shetty A.P., Vijayaraghavan G. (2001). The natural history of child spinal tuberculosis. J. Bone Jt. Surg. Br..

[80] Lashkarbolouk N., Mazandarani M., Ilharreborde B. (2023). Understanding the management of pediatric spondylodiscitis based on existing literature: A systematic review. BMC Pediatr..

[81] Rivas-Garcia A., Sarria-Octavio de Toledo L., Torrdelflores L., Beltran A., Baguena M.J. (2013). Imaging findings of spinal tuberculosis in children. Eur. J. Radiol..

[82] Garg R.K., Malhotra H.S., Jain A. (2019). Management of pediatric spinal tuberculosis: A narrative review. Neurol. India.

[83] Abel L., Fellay J., Haas D.W. (2018). Genetics of human susceptibility to active and latent tuberculosis: Present knowledge and future perspectives. Lancet Infect. Dis..

[84] Basu Roy R., Whittaker E., Seddon J.A. (2019). Tuberculosis susceptibility and protection in children. Lancet Infect. Dis..

[85] Bustamante J., Boisson-Dupuis S., Abel L., Casanova J.L. (2014). Mendelian susceptibility to mycobacterial disease: Genetic, immunological, and clinical features of inborn errors of IFN-γ immunity. Semin. Immunol..

[86] Al-Muhsen S., Casanova J.L. (2008). The genetic heterogeneity of Mendelian susceptibility to mycobacterial diseases. J. Allergy Clin. Immunol..

[87] Richards S., Aziz N., Bale S., Bick D., Das S., Gastier-Foster J., Grody W.W., Hegde M., Lyon E., Spector E. (2015). Standards and guidelines for the interpretation of sequence variants: A joint consensus recommendation of the American College of Medical Genetics and Genomics and the Association for Molecular Pathology. Genet. Med..

[88] Seddon J.A., Hesseling A.C., Godfrey-Faussett P., Schaaf H.S. (2012). Multidrug-resistant tuberculosis in children: Progress and challenges. Lancet Infect. Dis..

[89] World Health Organization (2025). WHO Consolidated Guidelines on Tuberculosis: Module 4: Treatment—Drug-Susceptible Tuberculosis Treatment.

[90] Carazo Gallego B., Moreno-Pérez D., Nuñez Cuadros E. (2016). Paradoxical reaction in immunocompetent children with tuberculosis. Int. J. Infect. Dis..

[91] Dash N., Jain L., Malik M. (2024). Paradoxical reaction to tuberculosis therapy among HIV-negative children: A systematic review and meta-analysis. Am. J. Trop. Med. Hyg..

