# Supplementary material for: An Unexpected Limp: A Pediatric Case of Pott’s Disease and a Literature Review
Source: Pathogens. 2026 Aug 31;15(9):919. doi: 10.3390/pathogens15090919 (PMC13610089; doi:10.3390/pathogens15090919)
Supplement: Supplementary file 1 [file pathogens-15-00919-s001.zip › pathogens-4473692-supplementary.pdf]

Table S1. Overview of relevant data collected from each study. M "male"; F "female"; C "cervical"; T "thoracic"; L "lumbar"; S "sacral"; NP "not performed"; NR "not reported"; PCR "polymerase chain reaction"; ZN "Ziehl-Neelsen staining"; H "isoniazid"; "R" rifampicin; "Z" pyrazinamide; "E" ethambutol".

Author, Year, Country	Sex, Age (years)	Spine Segment Affected	Number of vertebrae affected	XR/MRI/CT Findings	Positive Microbiology and/or Histology Indicative of TB	Medical Treatment	Surgical Treatment	Outcome
Bozzola et al. [8] 2013 Italy	M 13	T; L; S	6	Vertebral body destruction; paravertebral abscess; spinal cord compression	PCR (abscess drainage)	HRZ	Yes	Recovered
Hugar et al. [9] 2013 India	F 13	C	1	NP	ZN (abscess drainage)	NP	No	Death
Idris et al. [10] 2010 Nigeria	F 13	C; T	7	Vertebral body destruction; retropharyngeal abscess	ZN (abscess drainage)	HRZE	No	Death
Goh JLK et al. [11] 2018 China	F 2	T	3	Vertebral body destruction; paravertebral abscess	ZN, PCR (abscess drainage)	HRZE	Yes	Recovered
Kapoor et al. [12] 2007 India	F 0,7	C	1	Vertebral body destruction; paravertebral abscess; spinal cord compression	Histology Indicative of MTB (bone biopsy)	HRZE	Yes	Recovered
Diom et al. [13] 2011 Senegal	M 7	C	1	Vertebral body destruction; parapharyngeal abscess	ZN (abscess drainage)	HRZ	Yes	Recovered
Dagli et al. [14] 2009 Turkiye	M 17	T	1	Vertebral body destruction; paravertebral abscess	ZN, culture (abscess drainage)	HRZE	Yes	Recovered
Mohta et al. [15] 2017 India	F 16	T	3	Vertebral body destruction; paravertebral abscess	ZN, PCR (abscess drainage)	HRZE	Yes	Recovered
Aktürk et al. [16] 2015 Turkiye	M 16	L; S	3	Vertebral body destruction; psoas abscess	Culture (abscess drainage)	HRZE	Yes	Recovered
Parent et al. [17] 2024 Canada	M 15	C	1	Vertebral body destruction; paravertebral abscess; spinal cord compression	PCR, culture (sputum)	HRZE	No	Recovered
Chilundo et al. [18] 2023 Mozambique	M 3	L	2	Vertebral body destruction	Absent	HRZE	No	Recovered

Boussetta et al. [19] 2020 Tunisia	F 12	C; T	2	Vertebral body destruction; paravertebral abscess	Histology Indicative of MTB (bone biopsy)	HRZE	Yes	Recovered
Cheung et al. [20] 2012 Hong Kong	M 15	L	2	Vertebral body destruction; paravertebral abscess	PCR (abscess drainage)	HRZE; corticosteroids	Yes	Recovered
Yigit et al. [21] 2016 Turkiye	F 16	T	3	Vertebral body destruction; paravertebral abscess	Absent	HRZE	No	Recovered
Cassimos et al. [22] 2009 Greece	F 2	T	1	Vertebral body destruction; paravertebral abscess	NR	HRZE	No	Recovered
Khattry et al. [23] 2007 India	M 14	T; L	6	Vertebral body destruction; paravertebral abscess; spinal cord compression	Histology Indicative of MTB (bone biopsy)	HRZE	Yes	Recovered
Varo et al. [24] 2019 Mozambique	M 4	T; L	4	Vertebral body destruction; paravertebral abscess	Culture (abscess drainage)	HRZE	Yes	Recovered
Verma et al. [25] 2012 India	F 17	C	3	Vertebral body destruction; paravertebral abscess	NR	HRZE	No	Recovered
Taccari et al. [26] 2019 Italy	M 13	L	2	Vertebral body destruction; paravertebral abscess	PCR, culture (skin swab)	HRZE	No	Recovered
Singh et al. [27] 2016 India	M 0,7	C	1	Vertebral body destruction; paravertebral abscess	NR	HRZE	No	Recovered
Ateş et al. [28] 2011 Turkiye	F 16	T	1	Vertebral body destruction; paravertebral abscess	Culture (abscess drainage)	HRZE	Yes	Recovered
Leos-Leija et al. [29] 2022 Mexico	F 0,9	T	4	Vertebral body destruction; paravertebral abscess	ZN, PCR (bone biopsy)	HRZE	Yes	Recovered
Albarrak et al. [30] 2021 Saudi Arabia	F 10	T	3	Vertebral body destruction; paravertebral abscess; spinal cord compression	Culture (abscess drainage)	HRZE; corticosteroids	Yes	Recovered
Shah et al. [31] 2017 India	M 6	L	3	Vertebral body destruction; paravertebral abscess	PCR, culture (abscess drainage)	H, moxifloxacina, clofazimina, linezolid, amoxicillin/clavulanatE, PAS, Cm	Yes	Recovered
Wang et al. [32] 2020 China	M 17	T; S	4	Vertebral body destruction	PCR (lymph node biopsy)	HRZE	Yes	Recovered

Song et al. [33] 2010 South Korea	F 3	C; T	6	Vertebral body destruction; paravertebral abscess; spinal cord compression	Absent	HRZE	Yes	Recovered
Nakamura et al. [34] 2025 Japan	F 13	T	2	Vertebral body destruction; paravertebral abscess; spinal cord compression	ZN, PCR (bone biopsy)	HRZE; corticosteroids	Yes	Recovered
Das et al. [35] 2021 India	M 12	T	2	Vertebral body destruction; paravertebral abscess; spinal cord compression	PCR, culture (abscess drainage)	Z, E, levofloxacin, kanamycin, ethionamide, cycloserine	Yes	Recovered
Tian et al. [36] 2013 China	M 2	C	1	Vertebral body destruction; paravertebral abscess; spinal cord compression	ZN (abscess drainage)	HRZ	Yes	Recovered
Siwele et al. [37] 2019 South Africa	M 9	T; L	2	Vertebral body destruction; paravertebral abscess; spinal cord compression	Culture (abscess drainage)	HRZE, moxifloxacin, ethionamide, amikacin, pyrazinamide, terizidone	Yes	Recovered
Pike et al. [38] 2005 Canada	M 1,9	C; T	5	Vertebral body destruction; paravertebral abscess	Culture (gastric aspirate)	HRZ	Yes	Recovered
Goh et al. [39] 2016 Malaysia	F 2	T	2	Vertebral body destruction	Histology Indicative of MTB (bone biopsy)	HRZE	Yes	Recovered
Zawadzka et al. [40] 2025 Poland	F 10	T	3	Vertebral body destruction; paravertebral abscess; spinal cord compression	PCR (lymph node biopsy)	HRZE	Yes	Recovered
Imakiire et al. [41] 2020 Japan	F 3	C	1	Vertebral body destruction; paravertebral abscess; spinal cord compression	Culture (bone biopsy)	HRZE	Yes	Recovered
Masavkar et al. [42] 2012 India	M 4	L	1	Vertebral body destruction; paravertebral abscess	Culture (abscess drainage)	HRZE	Yes	Recovered
Alawad et al. [43] 2013 Sudan	M 6	C	2	Vertebral body destruction; retropharyngeal abscess	ZN (abscess drainage)	HRZ	Yes	Recovered
Emel et al. [44] 2006 Turkiye	F 17	T; L	4	Vertebral body destruction; paravertebral abscess	Histology Indicative of MTB (lymph node biopsy)	HRZE	Yes	Recovered
Cucuzza et al. [45] 2018	F 14	L; S	3	Vertebral body destruction; paravertebral	PCR, culture (abscess drainage)	HRZE	Yes	Recovered

Italia				abscess				
Lee et al. [46] 2010 Taiwan	M 1	C; T	5	Vertebral body destruction; paravertebral abscess	ZN (abscess drainage)	HRZ, streptomycine	Yes	Recovered
Van Well et al. [47] 2007 Holland	M 14	L	1	Vertebral body destruction; paravertebral abscess; spinal cord compression	PCR, culture (abscess drainage)	HRZE	Yes	Recovered
Giridharan et al. [48] 2020 India	M 2	T	2	Vertebral body destruction; paravertebral abscess; spinal cord compression	PCR, culture (abscess drainage)	Z, E, levofloxacin, ethionamide, cycloserine, kanamycina; corticosteroids	Yes	Recovered
Senanayake et al. [49] 2014 Sri Lanka	F 10	C	1	Vertebral body destruction; paravertebral abscess	NR	HRZE	No	Recovered
Basnet et al. [50] 2024 Nepal	F 2	T	NR	Vertebral body destruction; paravertebral abscess	Absent	HRZE; corticosteroids	No	Recovered
Sunday et al. [51] 2023 Nigeria	F 2	L	3	Vertebral body destruction	Absent	HRZE	No	Recovered
Bayhan et al. [52] 2015 Turkiye	M 1,7	T; L	4	Vertebral body destruction; paravertebral abscess	ZN, culture (abscess drainage)	HRZE	Yes	Recovered
Aouraghe et al. [53] 2023 Morocco	F 10	C; T	2	Vertebral body destruction; paravertebral abscess	PCR (sputum)	HRZE	No	Recovered
Haghighatkhah et al. [54] 2015 Iran	F 5	T; L	2	Vertebral body destruction; paravertebral abscess	PCR (bone biopsy)	HRZ	No	Recovered
Shukla et al. [55] 2011 India	M 5	L	2	Vertebral body destruction	ZN (gummas drainage)	HRZE	No	Recovered
Papan et al. [56] 2017 Germany	M 16	T	NR	Vertebral body destruction; paravertebral abscess	ZN, PCR (abscess drainage)	HRZE	Yes	Recovered
Osguthorpe et al. [57] 2001 United States	M 2	T	9	Vertebral body destruction; paravertebral abscess	Culture (abscess drainage)	HZE, streptomycin	Yes	Recovered
Oberdorfer et al. [58] 2012 Thailand	M 3	L	3	Vertebral body destruction	Absent	HRZE, ofloxacyn	Yes	Recovered
Ochoa et al. [59] 2006 Peru	M 3	T	5	Vertebral body destruction; paravertebral abscess	ZN (abscess drainage)	HRZ, streptomycin; corticosteroids	Yes	Recovered

Kiymaz et al. [60] 2006 Turkiye	F 1	T	5	Vertebral body destruction; paravertebral abscess; spinal cord compression	Culture (abscess drainage)	HRE	Yes	Recovered
Handryastuti et al. [61] 2024 Indonesia	M 16	T	4	Vertebral body destruction; paravertebral abscess	PCR (sputum)	HRZE; corticosteroids	Yes	Recovered
Posner et al. [62] 2010 United States	F 13	T	3	Vertebral body destruction; paravertebral abscess; spinal cord compression	ZN (abscess drainage)	HRZE	Yes	Recovered
Locham et al. [63] 2001 India	F 10	C	2	Vertebral body destruction; paravertebral abscess; spinal cord compression	NR	HRZE	No	Recovered
Avadhani et al. [64] 2010 India	M 14	L	2	Vertebral body destruction; paravertebral abscess	Culture (bone biopsy)	HRZE	Yes	Recovered
Afzal et al. [65] 2006 Pakistan	M 1,5	L	2	Vertebral body destruction; psoas abscess	Absent	HRZE	Yes	Recovered
Ferris et al. [66] 2020 Uinted States	F 10	T	4	Vertebral body destruction; paravertebral abscess	PCR, culture (abscess drainage)	HRZE	Yes	Recovered
Sutantoyo et al. [67] 2022 Indonesia	M 17	C; T; L	13	Vertebral body destruction; paravertebral abscess; spinal cord compression	PCR (bone biopsy)	HRZE	Yes	Recovered
Manoharan et al. [68] 2013 Great Britain	F 11	C; T	6	Vertebral body destruction; paravertebral abscess; spinal cord compression	Culture (abscess drainage)	HRZE	Yes	Recovered
Tufan et al. [69] 2005 Turkiye	M 0,7	T	2	Vertebral body destruction; paravertebral abscess	Culture (bone biopsy)	HRZE	Yes	Recovered
Garg et al. [70] 2009 India	F 10	C	2	Vertebral body destruction; retropharyngeal abscess	ZN, culture (abscess drainage)	HRZE	Yes	Recovered
Soeroso et al. [71] 2018 Indonesia	M 12	T	2	Vertebral body destruction; paravertebral abscess	NR	HRZE	No	Recovered
